# Effect of low sodium and high potassium diet on lowering blood pressure and cardiovascular events

**DOI:** 10.1186/s40885-023-00259-0

**Published:** 2024-01-02

**Authors:** Byung Sik Kim, Mi-Yeon Yu, Jinho Shin

**Affiliations:** 1 Division of Cardiology, Department of Internal Medicine, Hanyang University Guri Hospital, Guri, South Korea; 2 Division of Nephrology, Department of Internal Medicine, Hanyang University College of Medicine, Seoul, South Korea; 3 Division of Cardiology, Department of Internal Medicine, Hanyang University Medical Center, Hanyang University College of Medicine, 222, Wangsimni-ro, Sungdong-gu, Seoul, 04763 South Korea

**Keywords:** Sodium, Potassium, Blood pressure, Hypertension, Cardiovascular event

## Abstract

Incorporating aggressive lifestyle modifications along with antihypertensive medication therapy is a crucial treatment strategy to enhance the control rate of hypertension. Dietary modification is one of the important lifestyle interventions for hypertension, and it has been proven to have a clear effect. Among food ingredients, sodium and potassium have been found to have the strongest association with blood pressure. The blood pressure-lowering effect of a low sodium diet and a high potassium diet has been well established, especially in hypertensive population. A high intake of potassium, a key component of the Dietary Approaches to Stop Hypertension (DASH) diet, has also shown a favorable impact on the risk of cardiovascular events. Additionally, research conducted with robust measurement methods has shown cardiovascular benefits of low-sodium intake. In this review, we aim to discuss the evidence regarding the relationship between the low sodium and high potassium diet and blood pressure and cardiovascular events.

**Graphical Abstract:**

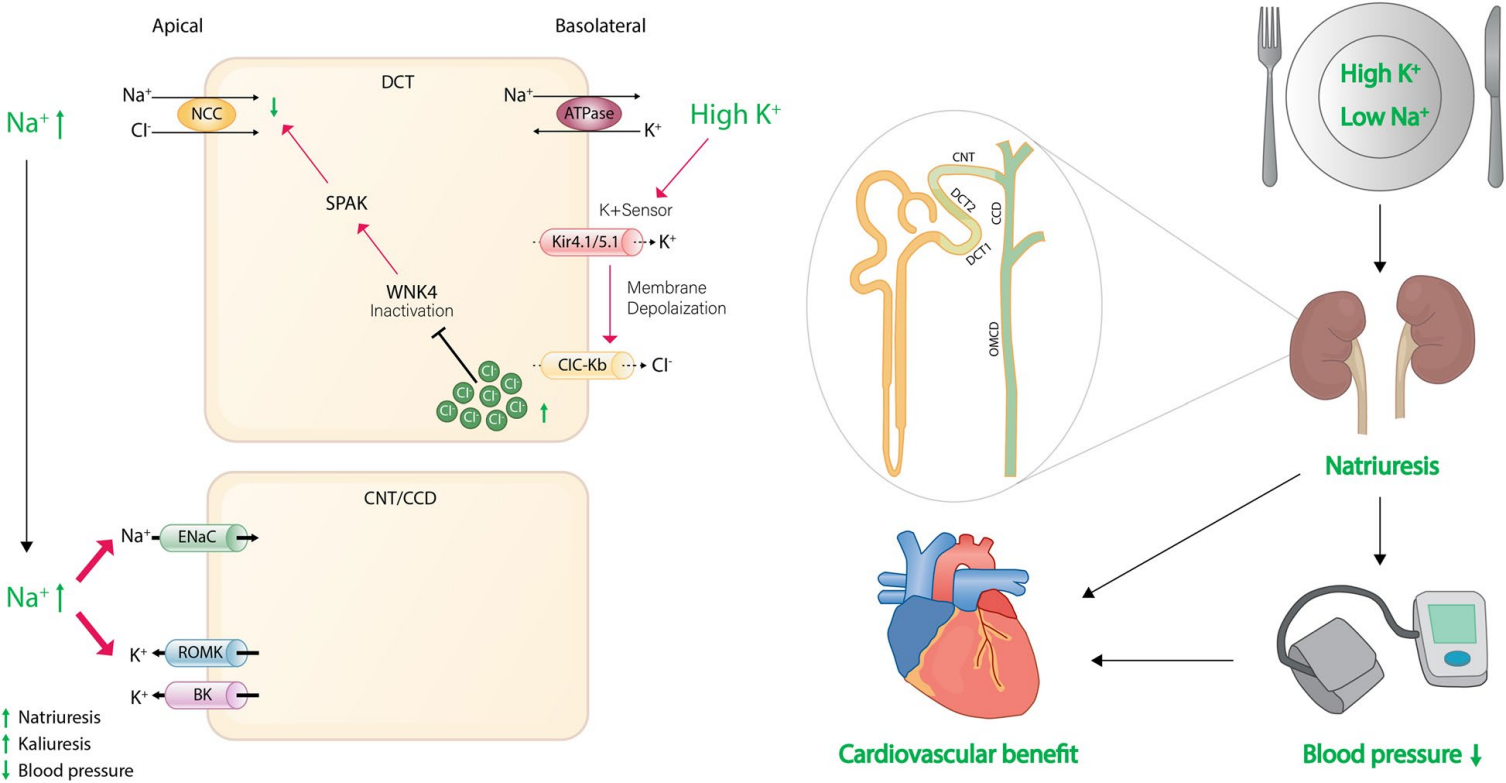

## Background

Hypertension is a major risk factor for cardiovascular events and deaths worldwide [1]. Despite the improvement in treatment strategies, including medications for hypertension, the control rate of hypertension has not reached 50% and remains an unmet challenge for public health [2, 3]. Implementing aggressive lifestyle modifications in addition to antihypertensive medication therapy is a very important treatment strategy for improving the control rate of hypertension [4, 5]. Dietary modification is one of the important lifestyle interventions for hypertension, and it has been proven to have a clear effect. Sodium and potassium are the most closely related elements to blood pressure among food ingredients [5]. Sodium is the ion with the highest concentration in extracellular fluid and determines the oncotic pressure of extracellular fluid, whereas potassium is the ion with the highest concentration inside cells and can indirectly affect blood pressure by participating in the renal excretion of sodium rather than directly regulating blood pressure [6]. As the population ages and the number of obese individuals continues to increase, the importance of dietary modification in blood pressure control is expected to increase [7].

Therefore, this paper aims to review the blood pressure-lowering effect of low sodium and high potassium diets and the latest clinical applications. “Salt” usually refers to sodium chloride, but for clarity in this paper, the term “sodium” is used uniformly except for specific terms. From the perspective of ion concentration between intracellular and extracellular fluid, the ion concentration of extracellular fluid is a more accurate expression than that of plasma ion concentration. However, since there is no significant difference in ion concentration between extracellular fluid and plasma, this paper considers that the concentration of ions without additional modifiers refers to the ion concentration in both extracellular fluid and plasma.

## Effects of sodium on blood pressure

### Sodium, extracellular fluid balance, and blood pressure

Sodium is an element that forms the highest concentration in extracellular fluid, and it exists in the form of sodium cations in body fluids, determining the osmotic pressure of extracellular fluid [7]. A constant osmotic pressure of extracellular fluid is an essential element for the survival, and in clinical practice, it is maintained steadily as long as water intake and excretion are not disrupted. If observed in very short time period, an increase in sodium intake may cause an increase in the osmotic pressure of the extracellular fluid and the phenomenon of the osmoreceptor cells in the intracellular fluid being stimulated by the movement of water out of the cells, causing thirst. However, this process is a dynamic process, and changes in osmotic pressure can only be observed as an increase in extracellular fluid, and it is difficult to observe an actual increase in sodium concentration [8]. Therefore, when sodium is consumed, only extracellular fluid increases without change in sodium concentration, meaning that sodium intake implies an increase in extracellular fluid. Conversely, decreasing sodium intake means decreasing extracellular fluid.

In the human body, the extracellular fluid is divided into two compartments: the interstitial fluid, which constitutes about 3/4 of the extracellular fluid, and the intravascular fluid, that is plasma volume, which constitutes about 1/4 of the extracellular fluid. Approximately every 3 hours, extracellular fluid is completely circulated once, and in this exchange process, about 180 L of kidney filtration is possible every day [9]. The human kidney is comprised of over a million functional units called nephrons. Blood enters the kidney through the afferent arteriole and is filtered in a network of small blood vessels known as the glomerulus, which deposits the filtrate into Bowman’s capsule - the initial component of the nephron. The glomerular filtrate then passes through the proximal tubule, the loop of Henle (with its descending and ascending limbs), the distal tubule, and the collecting duct. Approximately 65% of the sodium that enters the nephrons is reabsorbed from the renal tubules into the interstitial fluid and then into the general circulation through peritubular capillaries. The ascending limb of the loop of Henle actively secretes sodium into the interstitial space but is impermeable to water, resulting in the interstitial space between the ascending and descending limbs being hypertonic. This hypertonicity establishes an osmotic gradient between the fluid within the descending limb and the interstitial space, which removes water from the descending limb and increases the fluid tonicity within it. As the ascending limb is impermeable to water, sodium continues to be actively pumped out. Thus, the kidney is specialized according to the location, allowing for fine control of water and sodium through differentiation of sodium concentration depending on the location [10–12]. The rate of natriuresis depends on the ratio between the glomerular filtration rate, sodium tubular reabsorption, and secretion of the fluid containing sodium into the nephron tubular lumen [13].

As sodium intake increases, extracellular fluid volume increases, and intravascular volume increases in proportion. As a result, cardiac output increases, leading to a subsequent increase in blood pressure. The physiological mechanism by which an increase in BP in the renal arteries leads to an increased salt and water excretion is called pressure natriuresis [14].

### Salt sensitivity and blood pressure

In human body, the efficiency of sodium excretion in response to an increase in blood pressure varies greatly from individual to individual. For example, if there is a slight increase in blood pressure due to an increase in extracellular fluid, but if the excess fluid is immediately excreted through the kidneys and blood pressure returns to normal, it may appear in a clinical setting that blood pressure does not increase even with high sodium intake. On the other hand, if blood pressure slightly increases after an increase in extracellular fluid, and if the fluid or sodium cannot be excreted through the kidneys until a significantly high blood pressure is maintained, blood pressure will remain high until all the excess extracellular fluid is excreted after sodium intake. If the increased extracellular fluid is not normalized and another high sodium intake is consumed before blood pressure is fully recovered, blood pressure may continue to rise and be diagnosed as sustained blood pressure elevation. Thus, the degree of increase in blood pressure due to the increase in extracellular fluid following the intake of sodium or administration of sodium-containing fluids is defined as salt sensitivity [15].

In normal individuals, half of the ingested sodium is excreted within a day, and after 3–4 days, the total amount of sodium returns to its original level [16]. The degree of blood pressure elevation at this time is determined by individual salt sensitivity. The daily urinary sodium excretion slope (k_Na_, day^−1^) of 0.79 in normal individuals may decrease in the elderly and in patients with chronic kidney disease, which may result in a longer period for extracellular fluid to return to its original level than 3–4 days [6]. Therefore, if sodium is repeatedly ingested before recovery, patients with high salt sensitivity are at a higher risk of being diagnosed with hypertension [17]. The mechanisms that affect salt sensitivity include activation of the renin-angiotensin system, activation of the sympathetic nervous system, dysfunction of the renal sodium channel, and impaired endothelial cell function [18]. In clinical practice, important conditions related to salt sensitivity include aging, chronic kidney disease, and metabolic disorders including abdominal obesity that are closely related to these mechanisms [19].

### Sodium in human tissues

One of the mechanisms explaining salt sensitivity is that if the sodium consumed is present in a concentrated form in the extracellular fluid, even salt-sensitive patients may experience a significant temporal delay in the rise of blood pressure until the point at which the sodium storage capacity is reached, despite sodium intake. Until recently, it has been reported that sodium present in the skin or muscle can exist in a concentrated form, separate from the principle of osmotic pressure, to enable this buffering action [20]. However, some recent studies have also reported that the osmotic pressure of sodium present in the skin is not higher than that of the extracellular fluid, suggesting that the high concentration of sodium in the skin may be reflecting only subclinical tissue edema [21].

## Effects of potassium on blood pressure

### Regulation of potassium balance

Potassium is an electrolyte unrelated to osmotic pressure, and unlike sodium, there is no immediate mechanism to maintain the concentration of extracellular fluid. The amount of potassium intake through food and the amount excreted through the kidneys, gastrointestinal tract, sweat, etc. determine balance state. Therefore, under same excretion conditions, the amount of potassium consumed through food can have an impact on the potassium concentration of extracellular fluid [22, 23].

### Effect of potassium on sodium balance

**Fig. 1:**
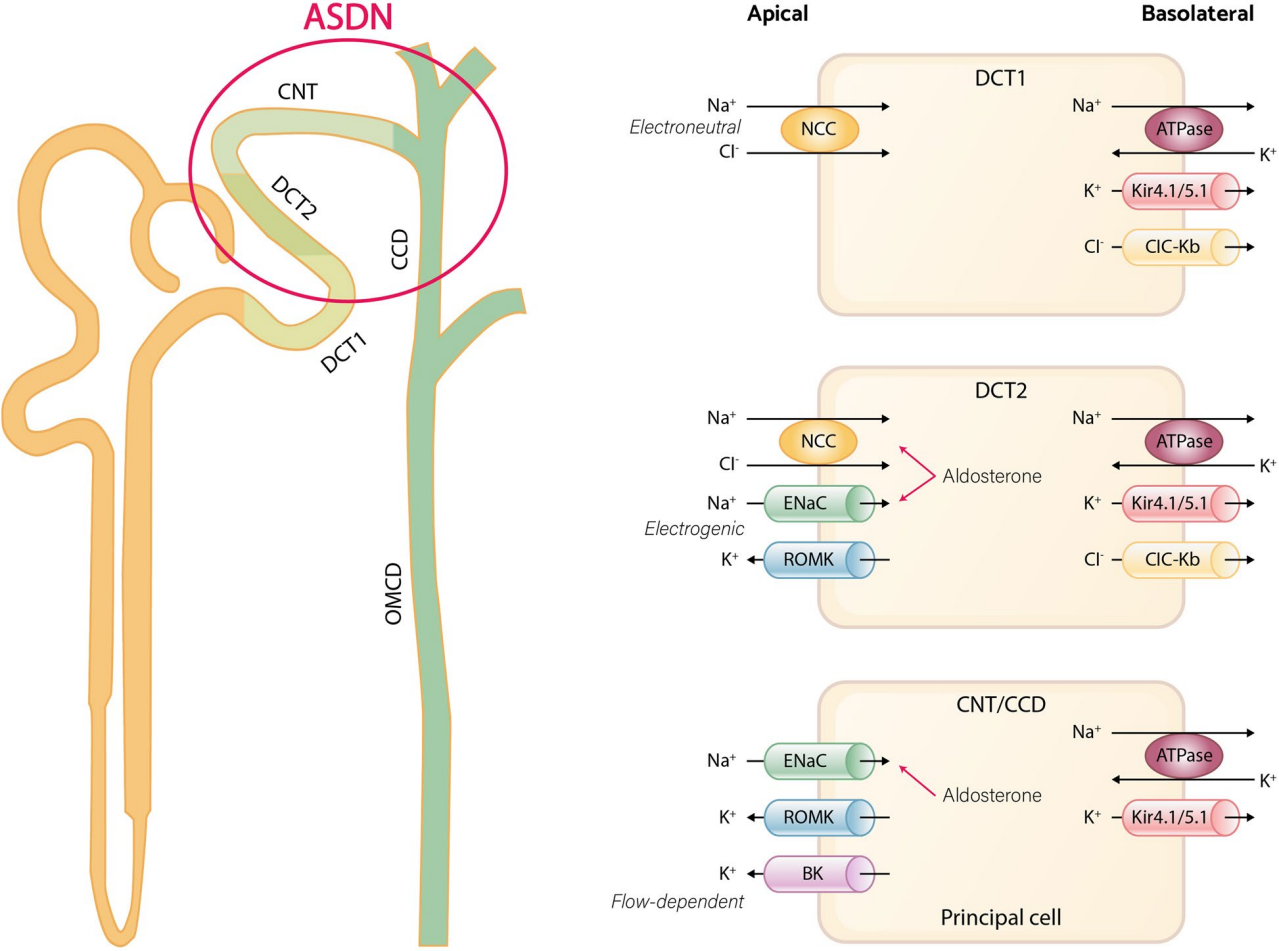
Overview of sodium and potassium handling in distal nephron

The intake of potassium has been reported to lower blood pressure in both human and animal models, and it is particularly effective when salt sensitivity is high or sodium intake is high [24, 25]. The aldosterone-sensitive distal nephron (ASDN) is responsible for the hormone-regulated unidirectional sodium transport and bidirectional potassium transport [26–28]. The ASDN consists of the late one-third of distal convoluted tubule (DCT2), the connecting tubule (CNT), and the cortical collecting duct (CCD). In the early two-thirds of the DCT (DCT1), the apical sodium-chloride cotransporter (NCC) is the major sodium transporter, which mediates electroneutral sodium reabsorption. In DCT2, NCC is co-expressed with the epithelial sodium channel (ENaC) and the renal outer medullary K+ (ROMK) channel [29, 30]. The main apical transporter responsible for electrogenic Na + reabsorption in the ASDN is ENaC [31]. The ENaC transporter orchestrates predominant electrogenic Na + reabsorption in the ASDN, generating a lumen-negative transepithelial voltage that ultimately plays a pivotal role in driving potassium secretion via ROMK. In addition to ROMK, the ASDN also expresses big-K+ channels (BK channels), which play a crucial role in flow-dependent potassium secretion [32]. This allows the ASDN to switch between electroneutral sodium reabsorption (via NCC) and electrogenic sodium reabsorption (via ENaC), enabling adjustments in urinary potassium and sodium excretion in response to dietary changes (Fig. 1).

**Fig. 2:**
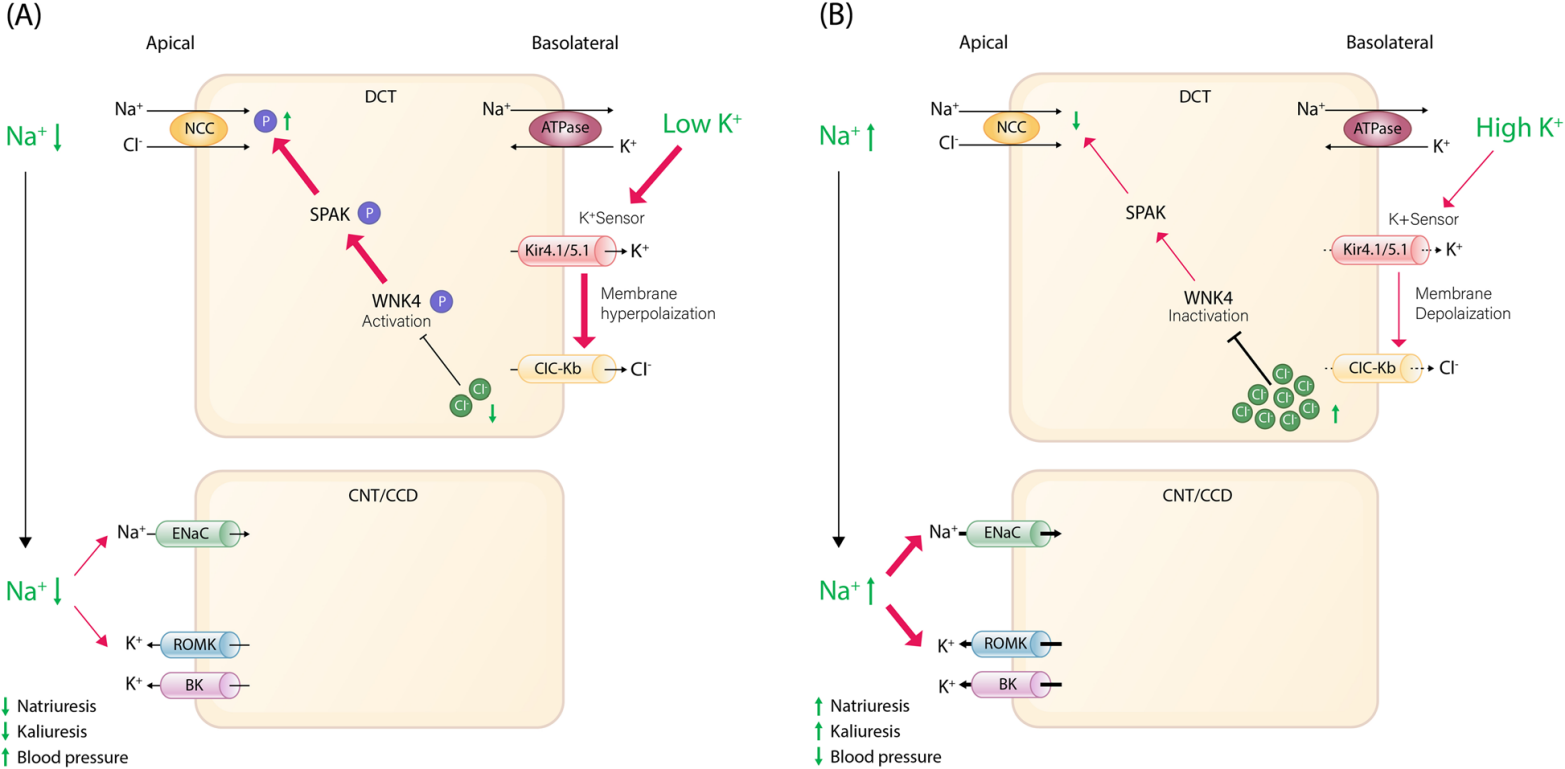
Role of the distal convoluted tubule as a potassium sensor regulating downstream potassium handling via sodium delivery in response to dietary potassium levels (**A**) effect of low potassium diet (**B**) effect of high potassium diet

Recent research indicates that the DCT acts as a potassium sensor and influences downstream potassium handling by regulating sodium delivery [33, 34]. In general, inward rectifier means hyperpolarization triggered potassium channel permit influx to stabilize the membrane potential. But in DCT, Kir4.1/Kir5.1 is the only potassium channel expressed on the basolateral membrane of the DCT so that it acts not as inward rectifier but as potassium leakage channel which is crucial in maintaining membrane potential [35]. It also has the role in detecting plasma potassium levels, and subsequently, modulating NCC activity [36]. Under conditions of low potassium diet, the potassium channel Kir4.1/Kir5.1 detects reduced extracellular potassium concentration, leading to potassium efflux through the basolateral plasma membrane of DCT cells [37, 38]. This process induces membrane hyperpolarization and stimulates chloride efflux [39]. The ensuing decrease in intracellular chloride concentration relieves the inhibition of chloride-sensitive kinases, especially with-no-lysine (K) kinases (WNKs), prompting autophosphorylation [40]. As a consequence, the activation of these phosphorylated WNKs triggers intermediate kinases such as Ste-20-related proline alanine-rich protein kinase (SPAK), which subsequently activate NCC, facilitating sodium reabsorption into the cell through NCC and leading to decreased natriuresis, kaliuresis, and elevated blood pressure (Fig. 2A) [41–43]. Salt-sensitive hypertension linked to NCC stimulation under low potassium intake [44]. Conversely, with a heightened potassium diet, Kir4.1/Kir5.1 channels are suppressed, leading to NCC dephosphorylation and diminished activity, subsequently decreasing sodium reabsorption. The suppression of NCC activity encourages kaliuresis while curbing sodium preservation, even in the face of heightened aldosterone levels. The kaliuretic effect resulting from dietary potassium intake actually precedes the rise in plasma aldosterone and is accompanied by natriuresis [3]. Moreover, due to the reduction in sodium reabsorption through NCC, there is an increased sodium delivery to the downstream ASDN. This intensifies the electrogenic sodium reabsorption mediated by ENaC, leading to the creation of an electrochemical gradient that propels the secretion of potassium through ROMK channels (Fig. 2B) [45–47]. Aldosterone plays a role in regulating urinary potassium excretion and sodium reabsorption by acting on the mineralocorticoid receptor at the late CNT and entire CCD and controlling the activity of genes involved in ENaC regulation [31, 48].

Dietary sodium affects NCC phosphorylation. High sodium intake restrains NCC phosphorylation, whereas low sodium intake stimulates it [49]. Despite high sodium intake, NCC activation due to a low potassium diet retains sodium, leading to elevated blood pressure. Conversely, under low sodium intake, a high potassium diet reduces NCC activity, even with activated systems [43, 50]. Jensen et al. noted that acute potassium loading prompts NCC dephosphorylation and natriuresis, regardless of sodium levels [45]. These findings emphasize prioritized potassium homeostasis over sodium regulation.

To conclude, the regulation of NCC activation is remarkably responsive to alterations in extracellular potassium levels [51, 52]. This sensitivity implies that even under conditions of elevated sodium intake, if extracellular potassium levels are also high, NCC activity is hindered, resulting in increased sodium excretion. This mechanism effectively counteracts blood pressure elevation, particularly beneficial for individuals with salt sensitivity increases in blood pressure [53, 54].

## Benefit of low sodium diet

### Recommendation of low sodium diet and issues related to interpretation of the evidence

There are many evidences that high sodium intake is associated with high blood pressure in both humans and animals [55–57]. Physiological requirements for sodium are < 500 mg/day in most healthy individuals, but currently usual sodium intake is between 3.5 and 5.5 g/day (which corresponds to 9–12 g of salt per day) in worldwide [58]. The World Health Organization (WHO) recommends a maximum daily intake of 2 g of dietary sodium (5 g of salt) for adults [59]. In addition, most countries and guidelines also suggest limiting intake to less than 2.0–2.4 g per day as part of a dietary approach to prevent high blood pressure and cardiovascular disease [5, 59–62]. Although there is general agreement regarding the reduction of dietary sodium intake in hypertensive patients and the general population, there are several issues related to the interpretation of the evidence supporting this approach. In the general population, there are claims that excessive restriction of sodium may not only have minimal benefits for lowering blood pressure, but may also increase blood cholesterol levels and the risk of mortality [63, 64]. On the other hand, some argue that a higher risk of mortality in low sodium intake group may be caused by reverse causality and estimation inaccuracy of sodium intake [65–67]. The other issues include whether there is repeated evaluation of sodium intake, the duration of the low sodium intervention period, and whether blood pressure reduction induced by sodium reduction is sustained or not [68–70].

### Assessment of sodium intake

Accurately measuring dietary sodium intake is crucial in individual and public healthy, but obtaining an unbiased measurement of sodium intake is challenging. Nutritional assessment tools, such as questionnaires, food diaries, and diet recalls, are often used in large nutrition surveys. However, these tools often exhibit inaccuracies when measuring individual dietary sodium intake compared to the gold standard of 24-hour urinary sodium excretion [71, 72]. The 24-hour urinary sodium excretion is widely regarded as the most accurate method of measurement of dietary sodium intake. It reflects about 90% of the sodium ingested over a day [73]. However, the accuracy can be affected by both undercollection and overcollection, as well as the high variability of sodium intake in an individual between days. Therefore, multiple collections are required to obtain a reliable estimation [74]. To overcome the methodological challenges of collecting complete 24-hour urine samples in large population-based studies, alternative estimation methods using spot urine have been introduced. Methods like Kawasaki, Tanaka, and International Cooperative Study on Salt, Other Factors, and Blood Pressure (INTERSALT), which estimate 24-hour urinary sodium excretion, have gained popularity in population-based studies [75–78]. However, these methods are not suitable for estimating salt intake in individuals due to their unreliability and inherent systematic bias [79]. Spot urine samples can lead to overestimation when sodium intake is low and underestimation when sodium intake is high [80]. This has been consistently observed in validation studies conducted to date, including the PURE Study [65, 81].

### Blood pressure-lowering effect of sodium restriction

Several studies restricting sodium intake have demonstrated a blood pressure-lowering effect. Recent meta-analyses have shown that effect of sodium reduction from an average high usual intake (4.6 g/day) to the recommended level (1.5 g/day) by the American Heart Association (AHA) was small in normotensive population (− 1.14/+ 0.01 mmHg in systolic/diastolic blood pressure). The effect of sodium reduction on blood pressure was pronounced decrease of − 5.71/− 2.87 mmHg in hypertensive population. The effect of sodium reduction was a little greater in Asian and black people than in white people [64]. In addition, the blood pressure-lowering effect of sodium reduction was more pronounced in patients with increased salt sensitivity, such as elderly, diabetic, metabolic syndrome or chronic kidney disease patients [82].

The blood pressure-lowering effect of sodium restriction is more effective in hypertensive patients than in normotensive patients, and even patients who are already receiving drug therapy can expect an additional 3 mmHg reduction in blood pressure by implementing sodium restriction, making it easier to reduce medication. Additionally, adding weight loss to sodium restriction can lead to an additional effect that is as significant as the blood pressure lowering effect of sodium restriction alone [83]. Moreover, sodium restriction can effectively reduce blood pressure even in patients with resistant hypertension [84]. In addition, excessive sodium intake is known to weaken the effect of renin-angiotensin system blockers [85].

### Cardiovascular benefit of sodium reduction

Although there was controversy in some UK Biobank studies [86–88], most large-scale cohort studies have shown an positive association between high sodium intake and cardiovascular events [78, 89–91]. However, large observational studies have not shown consistency regarding the relationship between low sodium intake and cardiovascular events. A meta-analysis of high-quality prospective cohort studies demonstrated a dose-dependent relationship between sodium intake and cardiovascular events [92, 93]. Whereas, certain large cohort studies, such as the Prospective Urban Rural Epidemiology (PURE) study, have identified a J-curve phenomenon in which an inverse association exists between low sodium intake and cardiovascular events [78, 94–96]. This J-curve phenomenon is thought to be hypothesized based on the role of sodium, which is known to play a critical role in normal human physiology [97], and the activation of the renin–angiotensin–aldosterone system, as well as the observed increase in the lipid profile with low sodium intake [64]. Although there has been a debate surrounding the explanation for the J-curve phenomenon, there is a growing consensus that much of the effect can be attributed to methodological artifacts (such as the estimation of sodium intake using spot urine samples) or epidemiological analyses (such as confounders and reverse causality) [98].

Several randomized controlled trials also have conducted to investigate the impact of salt reduction on cardiovascular disease, but the majority of these studies had inadequate sample sizes and durations. According to a previous Cochrane review, the evidence supporting the effectiveness of interventions aimed at reducing dietary salt on cardiovascular events was small [99, 100]. Contrary to these claims, opposing results have also been reported that reducing salt intake is associated with a significant decrease in cardiovascular events [101].. In addition, National Academy of Sciences (NAS) report concluded that reducing dietary sodium intake can prevent cardiovascular events based on a meta-analysis of well-designed relatively long-term trials, including the Trials of Hypertension Prevention (TOHP), and the Trials of Nonpharmacologic Intervention in the Elderly (TONE) [102–104]. More recently, a meta-analysis including the Health Professionals Follow-up Study (HPFS), the Nurses’ Health Study (NHS), NHS II, the Prevention of Renal and Vascular End-Stage Disease (PREVEND), TOHP I, and TOHP II trials, which repeatedly conducted 24-hour urine collection as the most appropriate method, reported a dose-dependent and significant association between high sodium intake and risk of cardiovascular events [83, 96, 105–108]. They reported that daily increment of 1 g in sodium excretion was associated with an 18% increase in cardiovascular events [93]. Table 1 summarizes large observational studies (more than 1000 sample) that have evaluated the association between sodium intake and cardiovascular events using 24-hour urinary sodium excretion [79, 93, 95, 109–113].

**Table 1:** Major observational studies evaluating the relationship between sodium intake and cardiovascular events through 24-hour urinary sodium excretion

Study (year)	Population	Estimation of sodium intake	Follow-up (years)	Outcomes	Result	Reference
Stolarz-Skrzypek et al. (2011)	3681 participants without CVD	24-hour urinary sodium excretion	7.9	CV death	Weak inverse association	[109]
Thomas et al. (2011)	2807 participants with type 1 DM	24-hour urinary sodium excretion	10.0	All-cause death	J-curve association	[110]
PREVEND (2014)	7543 participants without CVD	24-hour urinary sodium excretion	10.5	Coronary heart disease events	No association	[111]
Singer et al. (2015)	3505 participants with HTN	24-hour urinary sodium excretion	18.6	CV death and all-cause death	Direct association with all-cause death	[112]
Mills et al. (2016)	3757 participants with CKD	Multiple 24-hour urinary sodium excretion	6.8	Composite of CVD events	Linear association	[95]
Vuori et al. (2020)	4630 general population	24-hour urinary sodium excretion	14.0	Composite of CVD events	Direct association	[113]
TOHP I and II (2016)	3011 participants with prehypertension	Multiple 24-hour urinary sodium excretion	23.9 and 18.8	All-cause death	Linear association	[79]
Meta-analysis of HPFS, NHS I, NHS II, PREVEND, TOHP I, and TOHP II (2022)	10709 general population	Multiple 24-hour urinary sodium excretion	8.8	Composite of CVD events	Linear association	[93]

*CVD* cardiovascular disease, *CV* cardiovascular, *DM* diabetes mellitus, *PREVEND* the Prevention of Renal and Vascular End-Stage Disease, *HTN* hypertension, *CKD* chronic kidney disease, *TOHP* the Trials of Hypertension Prevention, *HPFS* the Health Professionals Follow-up Study, *NHS* the Nurses’ Health Study.

Due to the issues of high cost and time associated with conducting large randomized clinical trials (RCTs), relatively cost-effective cluster RCTs have been conducted using a method that compares potassium-rich salt substitutes with regular salt [114–116]. At present, the most representative large-scale cluster RCT is the Salt Substitute and Stroke Study (SSaSS) trial, which included 20,995 participants aged 60 years or older with a history of stroke or hypertension in 600 villages in rural China. In the SSaSS trial, the participants who received the salt substitute (75% sodium and 25% potassium) had significantly lower rates of cardiovascular events including death than those who received regular salt (100% sodium) [116]. There may be controversies about the design of the SSaSS trial, such as the absence of a placebo intervention and the inability to confirm whether the benefits in the intervention group were due to potassium supply. However, it cannot be denied that it provided important community-based evidence on low sodium diet.

When considering these pieces of evidence as a whole, it may be difficult to provide conclusive evidence due to challenging barriers. However, there is still controversy over the interpretation of observational study results, studies emphasizing the accurate measurement of sodium intake and intervention studies have demonstrated a positive association between sodium intake and blood pressure, as well as a direct linear association with cardiovascular events. Therefore, at this point, it is necessary to emphasize the daily sodium intake limit (less than 2.0–2.4 g per day) recommended by the WHO and clinical practice guidelines. In addition, reducing salt intake through population-wide interventions is likely to be a cost-effective choice in public health [117].

### Sustainability of sodium intake restriction

Despite these beneficial effects, it is quite difficult to continue a low sodium diet [118]. To achieve this, a systematic approach is crucial in accordance with the principle of evaluating the general treatment adherence rate of chronic diseases. In addition to the development and education of medical personnel for team-based approaches and appropriate therapeutic counseling to support individual patient behavioral change, considerations for social or ecological factors related to sodium restriction must also be included [119]. For example, it is known that in most cases, additional sodium beyond the natural sodium found in raw ingredients is supplied during food processing or commercial food sales. Therefore, it is important to reduce processed foods and check the sodium content indicated on food labels [120]. Food labeling can give motivation to consumers choose low sodium products [121]. Additionally, certain labeling practices can encourage manufacturers to reformulate their products to contain less sodium. In order to choose low sodium content, it is important to choose products without additional salt, reduce foods seasoned or pickled with salt or seasonings, use low sodium spices with spicy flavors, choose carefully when eating out, adjust the nutrient content of food, and avoid using sodium at the table. Consultation with a skilled nutritionist on behavior change can be helpful in implementing these methods. If sodium restriction is possible through social or institutional improvements, it can reduce the effort to consciously reduce sodium intake, thus maximizing efficiency [122]. Self-monitoring is crucial in patients’ self-management, particularly for chronic diseases such as diabetes, asthma, and heart failure [123]. The potential benefits of self-monitoring are promising, as literature indicates it may enhance self-management, symptom management, and disease regulation, resulting in fewer complications, improved coping and attitudes towards the illness, realistic goal setting, and an overall better quality of life [124, 125]. Self-blood pressure monitoring, such as home blood pressure, has been shown to improve treatment adherence in hypertension and is actively recommended [5, 126]. For example, when counseling patients on their home blood pressure measurement results, regularly showing patients with a few days of blood pressure increases can help them monitor the effects of sodium intake on blood pressure and can help them understand their salt sensitivity and the blood pressure-lowering effect of sodium restriction, which can have a positive effect on sustained behavior change.

## Benefit of high potassium diet

Unlike the controversy surrounding the effects of low sodium intake, there is relatively consistent evidence regarding the blood pressure-lowering and cardiovascular benefits of high potassium intake. The INTERSALT study found that potassium intake was an important independent determinant of blood pressure, independent from sodium intake [127]. In the 10-year follow-up study conducted in the Korean population, which had a relatively low sodium intake of approximately 2500 mg per day, there were concerns regarding the accuracy of the food frequency questionnaire. The study revealed that potassium intake and the Na:K ratio were more reliable predictors of cardiovascular outcomes [128]. The PURE study also reported that as urinary potassium excretion increased, systolic blood pressure decreased, and the rates of mortality and cardiovascular events were decreased [129]. Several meta-analyses have consistently shown similar results. In a meta-analysis comprising 33 randomized controlled trials (*N* = 2609), data were collected by comparing intervention and control groups in which the only variable was potassium supplementation. This meta-analysis revealed a significant reduction in systolic blood pressure of 3.1 mmHg and diastolic blood pressure of 2.0 mmHg in the groups that received potassium supplementation [130]. Similarly, a more recent meta-analysis conducted in 2013, including 22 randomized controlled trials and 11 cohort studies (*N* = 1606), found a systolic blood pressure reduction of 3.5 mmHg and diastolic blood pressure reduction of 2.0 mmHg in adults with hypertension who received potassium supplementation. However, this effect was not observed among individuals without hypertension. Furthermore, higher potassium intake was associated with a 24% reduced risk of stroke [131]. Similarly, a meta-analysis examined 10 prospective studies (*N* = 268,276 participants, 8695 stroke cases) that investigated the relative risks of stroke across three categories of potassium intake or with potassium intake as a continuous variable. This analysis revealed that for every 1 g/day increase in potassium supplementation, there was an 11% reduction in the relative risk of stroke [132]. These cardiovascular beneficial effects of potassium have been consistently observed in meta-analyses, including studies on 24-hour urinary potassium excretion [93].

In a SSaSS trial [116], the group that replaced regular salt with a salt-substitute consisting of 75% sodium chloride and 25% potassium chloride showed a reduced risk of major adverse cardiovascular events (risk ratio 0.87), death from any cause (risk ratio 0.88), death from vascular insult (risk ratio 0.87), nonfatal acute coronary syndrome (risk ratio 0.70), and stroke (risk ratio 0.86). It is important to note that the SSaSS trial excluded patients who were at risk of hyperkalemia, such as those on potassium-sparing diuretics, taking potassium supplements, or with advanced chronic kidney disease. This exclusion raises concerns about the potential risk of hyperkalemia in this specific population. In individuals with chronic kidney disease, the prevention and management of hyperkalemia can result in significant medical expenses and burdens on the healthcare system [133, 134]. Further research is required to determine the long-term effects of potassium supplementation in patients with chronic kidney disease.

## DASH diet and sodium potassium ratio

Potassium is abundant in vegetables, fruits, potatoes, sweet potatoes, and legumes and is an important component of the dietary approaches to stop hypertension (DASH). The recommended daily intake of potassium in the US is 4.7 g, while the WHO recommends a daily intake of 3.5 g [135, 136]. The DASH diet is a standard diet for lowering blood pressure in hypertensive patients. It showed a blood pressure lowering effect of 11 mmHg and 3 mmHg in hypertensive patients and normal individuals, respectively, and the effect is independent of sodium intake, with additional blood pressure-lowering effects expected when combined with a low sodium diet [137]. The DASH diet is widely recognized as the most effective dietary pattern for lowering blood pressure. Because the DASH diet is high in fruits, vegetables, and low-fat dairy products, it provides a means to enhance intake of potassium, calcium, magnesium, and fiber [138]. Potassium is mainly related to fruit and vegetable intake. The DASH diet typically provides 4.7 g of potassium per day, which is the same as the recommended daily intake of potassium [139]. A low sodium diet and DASH diet recommended for patients with hypertension can be summarized as a diet that reduces sodium and increases potassium, in other words, a diet that reduces the sodium-potassium ratio. It can be inferred that the sodium-potassium ratio may be more important in actual situations than the individual effects of sodium or potassium, which is consistent with the theoretical basis for sodium reabsorption discussed earlier. Although there are no established recommendations for the sodium-potassium ratio, WHO recommends a ratio of 1.0 based on mol units [59].

## Conclusions

In conclusion, increased sodium intake has been shown to have a positive correlation with blood pressure and cardiovascular events, especially in hypertensive population, in past studies conducted in various ways with sound methodology, particularly accurate measurements of salt intake. Reflecting this evidence, efforts to reduce sodium intake are already underway in many countries [140], and many clinical guidelines recommend reducing sodium intake [5, 62, 138, 141]. In addition, a high potassium diet also plays an important role in preventing and controlling hypertension and cardiovascular diseases. To achieve effective blood pressure control and reducing cardiovascular events, it is important to implement a low sodium diet and a high potassium diet simultaneously to maintain a low sodium-potassium ratio in the diet.

## Data Availability

Not applicable.

## Abbreviations

ASDN: aldosterone-sensitive distal nephron
DCT: distal convoluted tubule
CNT: connecting tubule
CCD: cortical collecting duct
NCC: sodium-chloride cotransporter
ENaC: epithelial sodium channel
ROMK: renal outer medullary K+ channel
BK: big-K+ channels
WNK: with-no-lysine (K) kinases
SPAK: Ste-20-related proline alanine-rich protein kinase
OSR1: oxidative stress-responsive kinase 1
WHO: World Health Organization
AHA: American Heart Association
NAS: National Academy of Sciences
TOHP: the Trials of Hypertension Prevention
TONE: the Trials of Nonpharmacologic Intervention in the Elderly
HPFS: the Health Professionals Follow-up Study
NHS: the Nurses’ Health Study
PREVEND: the Prevention of Renal and Vascular End-Stage Disease
RCT: randomized clinical trial
SSaSS: the Salt Substitute and Stroke Study
INTERSALT: the International Cooperative Study on Salt, Other Factors, and Blood
PURE: the Prospective Urban Rural Epidemiology
DASH: the dietary approaches to stop hypertension.
